# Image type reveals evolutionarily shaped perceptual and conceptual mechanisms of pareidolia

**DOI:** 10.1038/s41598-026-47242-x

**Published:** 2026-04-15

**Authors:** N. Göbel, M. Camenzind, T. Nef, U. P. Mosimann, R. M. Müri, A. K. Eberhard-Moscicka

**Affiliations:** 1 Department of Neurology, Inselspital, Bern University Hospital and University of Bern, Bern, Switzerland; 2 Research and Analysis Services, University Hospital Basel and University of Basel, Basel, Switzerland; 3 Gerontechnology and Rehabilitation Group, ARTORG Center for Biomedical Engineering Research, University of Bern, Bern, Switzerland; 4 Rehaklinik Zihlschlacht AG, Zihlschlacht, Switzerland

**Keywords:** Pareidolia, Visual perception, White noise images, Natural images, Evolutionary psychology, GESTALT psychology, Human behaviour, Object vision

## Abstract

Pareidolia—the perception of illusory patterns such as faces or objects in ambiguous stimuli—has been extensively studied, but less is known about how different visual environments shape this phenomenon. This study investigated pareidolia experienced in unmodified natural images (NI) and white noise (WN) images in 81 healthy participants under open-ended instructions. Across both conditions, animals were the most commonly perceived pareidolia, consistent with evolutionary pressures favouring rapid detection of biologically relevant stimuli. Importantly, the semantic categories of pareidolia differed by image type: NI evoked significantly more “Natural World” pareidolia, while WN images primarily elicited “Human-Created” pareidolia. This dissociation suggests that NI stimuli engage bottom-up perceptual organization processes whereas WN images depend more heavily on top-down semantic knowledge and mental imagery. Together, these findings indicate that stimulus type modulates the relative contribution of perceptual and conceptual processes in the resolution of visual ambiguity. Beyond their methodological implications for pareidolia research, these results carry broader relevance for understanding creative perception, hallucinations, and cognitive biases that may have been shaped by evolutionary pressures. They also underscore the need for careful and deliberate selection of visual stimuli when employing open-ended tasks in future studies.

**Supplementary Information:**

The online version contains supplementary material available at 10.1038/s41598-026-47242-x.

## Introduction

Pareidolia—the perception of meaningful images or patterns in ambiguous stimuli—is a universal perceptual phenomenon wherein observers interpret vague sensory input as familiar objects or forms^1,2^. Etymologically derived from the Greek *para* (παρά: beside, instead) and *eidōlon* (εἴδωλον: image, form), pareidolia manifests as e.g., perception of faces in clouds, animals in rock formations, or figures in tree bark. This phenomenon reflects the brain’s pattern-recognition mechanisms, which impose structure and meaning on sensory input even in the absence of genuine patterns.

From a psychological perspective, pareidolia intersects with Gestalt psychology and semantic memory systems. Gestalt theory^3,4^ posits that perception arises from holistic organization rather than from summation of discrete elements. The Gestalt principle of “closure”—the tendency to complete fragmented stimuli into coherent objects—facilitates pareidolia by driving the brain to resolve ambiguous inputs into recognizable forms. Similarly, "figure-ground perception” enables discrimination between focal objects and backgrounds, structuring ambiguous stimuli into interpretable configurations^3^.

Semantic knowledge—the long-term memory system encoding conceptual meaning, categorical relationships, and contextual associations^5^—provides the interpretive framework for pareidolia. When processing ambiguous stimuli, the brain retrieves stored semantic representations to assign meaning, resulting in pareidolic experiences. For example, recognizing a cloud formation as a “face” integrates visual processing with semantic knowledge of facial features, their spatial relationships, and associated social significance. Thus, pareidolia may emerge from the interaction between Gestalt organizational principles and semantic memory retrieval, demonstrating the brain’s tendency to impose meaningful interpretations on environmental stimuli.

Face pareidolia—the perception of faces in ambiguous or noise-containing stimuli—became a valuable paradigm in cognitive neuroscience, offering a controlled means of probing the interaction between perceptual and higher-order interpretive processes. Neuroimaging studies identified key neural networks involved in processing face pareidolia, including the right fusiform face area (rFFA) and an extended network encompassing frontal and occipito-temporal regions such as the prefrontal cortex (PFC), inferior temporal regions, and primary visual cortices (V1 and V2)^6,7^. Further work characterized the temporal dynamics of face pareidolia, showing that early responses (~ 100 ms) closely resemble those evoked by veridical faces, whereas by ~ 250 ms signals diverge, becoming indistinguishable from responses to ordinary objects^8^. These dynamics were interpreted as evidence for a broadly tuned face-detection mechanism prioritizing sensitivity over specificity—a system optimized to minimize misses at the cost of occasional false alarms. Converging behavioral evidence supports this account: pareidolic faces capture attention rapidly and robustly, even when inverted, indicating that their salience cannot be explained solely by low-level visual features^9^.

Further work highlights importance of top-down modulation. Perceptual learning enhances inferior temporal responses to degraded stimuli and increases connectivity with attention- and memory-related parietal regions, suggesting contributions from imagery and feature binding^10^. Brief pareidolic presentations also elicit face-selective activity^11^, reflecting rapid interactions between bottom-up signals and higher-order expectations. Ambiguous illusory faces elicit attenuated neural responses and slower detection when presented among veridical human faces, indicating interference between stimulus-driven and top-down face temporal templates^12^.

Individual differences also shape pareidolia propensity. Although all individuals display a general bias to perceive human features in ambiguous stimuli, those endorsing paranormal or religious beliefs adopt particularly liberal decision criteria, resulting in higher false-alarm rates^13^.

Experimentally, pareidolia has been induced using unmodified natural images (NI)^1,14^, Gaussian-blurred scenery^15^, degraded photographs^16^, white noise (WN) patterns^17^, or fractal cloud-like images^18^. However, whether stimulus type influences the frequency or categorical content of pareidolia remains unclear, and to date, no studies have directly compared the effects of different image types.

Top-down attentional mechanisms in vision are frequently triggered or modulated by explicit verbal instructions such as “search for faces” or “detect target objects” that establish specific expectations and elicit strategic, preparatory behaviors in participants. Such goal-directed instructions robustly engage endogenous top-down control, but they also introduce demand characteristics and systematically bias perceptual processing toward anticipated features or categories. By contrast, open instructions deliberately minimize externally imposed expectations and reduce strategic anticipation. This approach enables attentional deployment to emerge more spontaneously, guided predominantly by the intrinsic semantic content of the stimuli, participants’ pre-existing knowledge, personal experiences, and implicit assessments of relevance. As a result, open instruction provides a purer window into the dynamic interplay between bottom-up semantic processing and endogenous top-down influences, particularly under conditions where strong, externally cued task sets are absent. The dual-stimuli design (white noise images paired with natural photographs) under open instructions thus offers several key advantages: (1) It enables a direct within-subject contrast between conditions where bottom-up configural cues are minimal or absent (WN) versus rich and ecologically valid (natural scenes), highlighting the relative contributions of stimulus structure versus endogenous factors in driving pareidolia. (2) It reduces potential confounds arising from stimulus-specific habituation, expectation carry-over, or differential engagement across separate experiments. (3) It strengthens inferences about the generalizability of spontaneous attentional and perceptual mechanisms, as any observed patterns must transcend low-level stimulus differences.

To our knowledge, this is the first study to apply open instructions consistently throughout the entire experiment, using carefully neutral and non-directive phrasing for both white noise and natural photograph stimuli. Although free-viewing or passive observation paradigms have previously been used in pareidolia research to minimize overt task demands (e.g.,^8,14,19–21^), these approaches have typically been restricted to a single stimulus type—either visual noise or natural scenes—rather than systematically comparing both within the same participants and instructional framework.

## Participants and methods

**Fig. 1:**
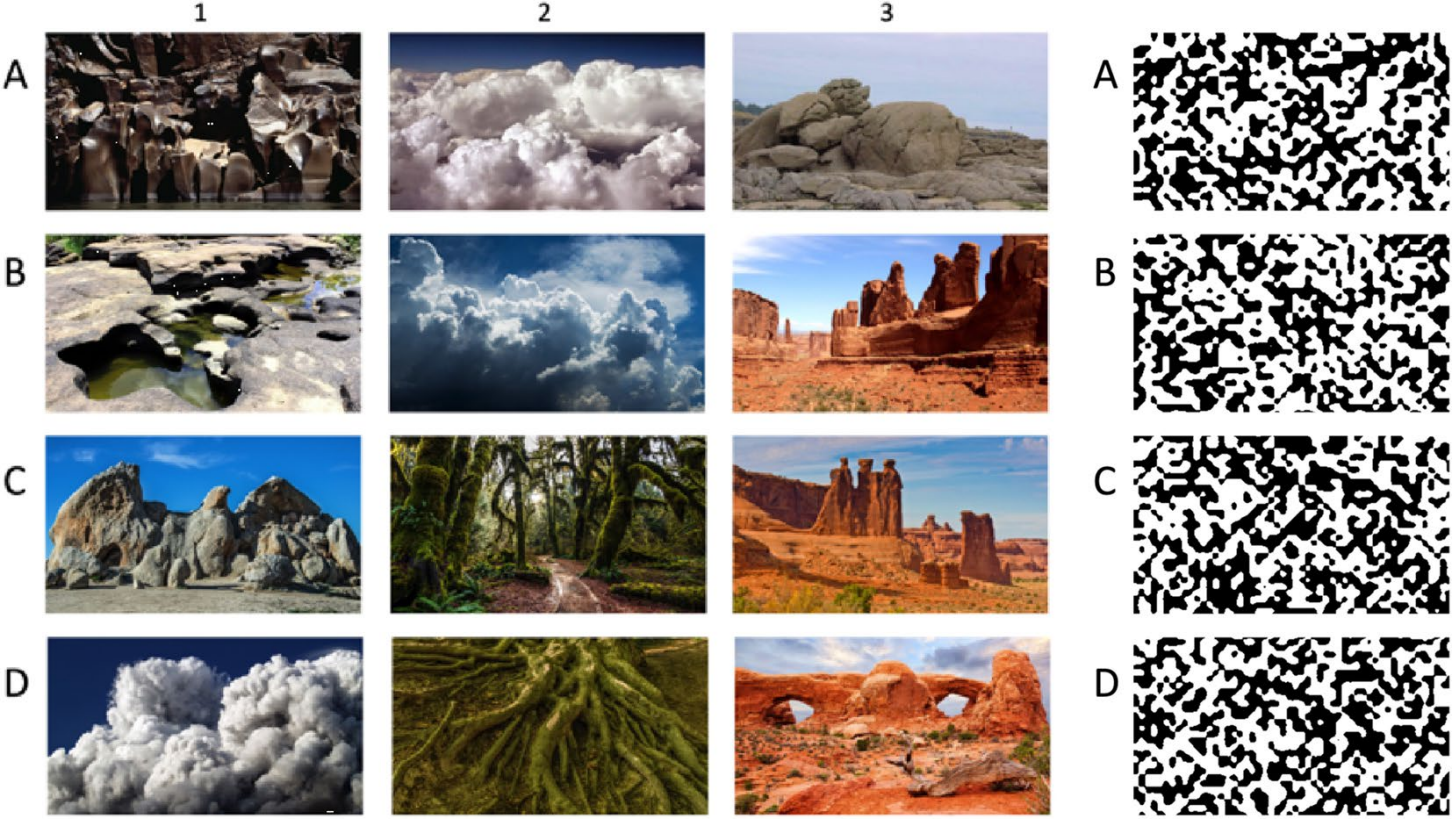
Natural (NI) and white noise (WN) images used in the study. Four series (A-D) of images were presented, with each series containing three NI images (left) and four WN images (right). The presentation order of the series was randomized across participants.

We report data of 81 healthy participants (mean age: 49.94 years, SD: 17.23 years, range: 20–78 years; mean years of education: 16.46, SD: 3.47; 10 left-handed, 47 females). For each participant, a series consisting of three natural images (NI) and one white noise image (WN) (see Fig. 1) was randomly selected to induce pareidolia. The images were presented in a randomized order, with each image shown for five minutes [cf.^1,14^]. The study was conducted in accordance with the principles outlined in the Declaration of Helsinki. Prior to commencement, the research protocol received approval from the local Ethics Committee of the Canton of Bern. All participants provided written informed consent before participating in the study.

The task was programmed using the development platform Unity 2019.3 (Unity Software Inc., San Francisco, United States), and the images were displayed on a Lenovo Yoga tablet 720-15IKB (15.6 Zoll, i7, 16 GB RAM). Participants were provided with a compatible digital pen (Lenovo active Pen) and were asked to draw the pareidolia they perceived in the presented images. The task instruction was “Look at the pictures and let your imagination run free. Draw everything you can see, (for NI: except the objects themselves) and say each time what it is.” Participant’s drawings were automatically saved on the tablet, while the oral descriptions of the drawn pareidolia were recorded using a voice recorder (Jabra PHS001U). These recording were subsequently transcribed by the examiners. Prior to the experiment, all participants were shown the same example of a natural image and a white noise image, each accompanied by two exemplary pareidolia, which were identical for all the participants.

### Statistical analysis

**Fig. 2:**
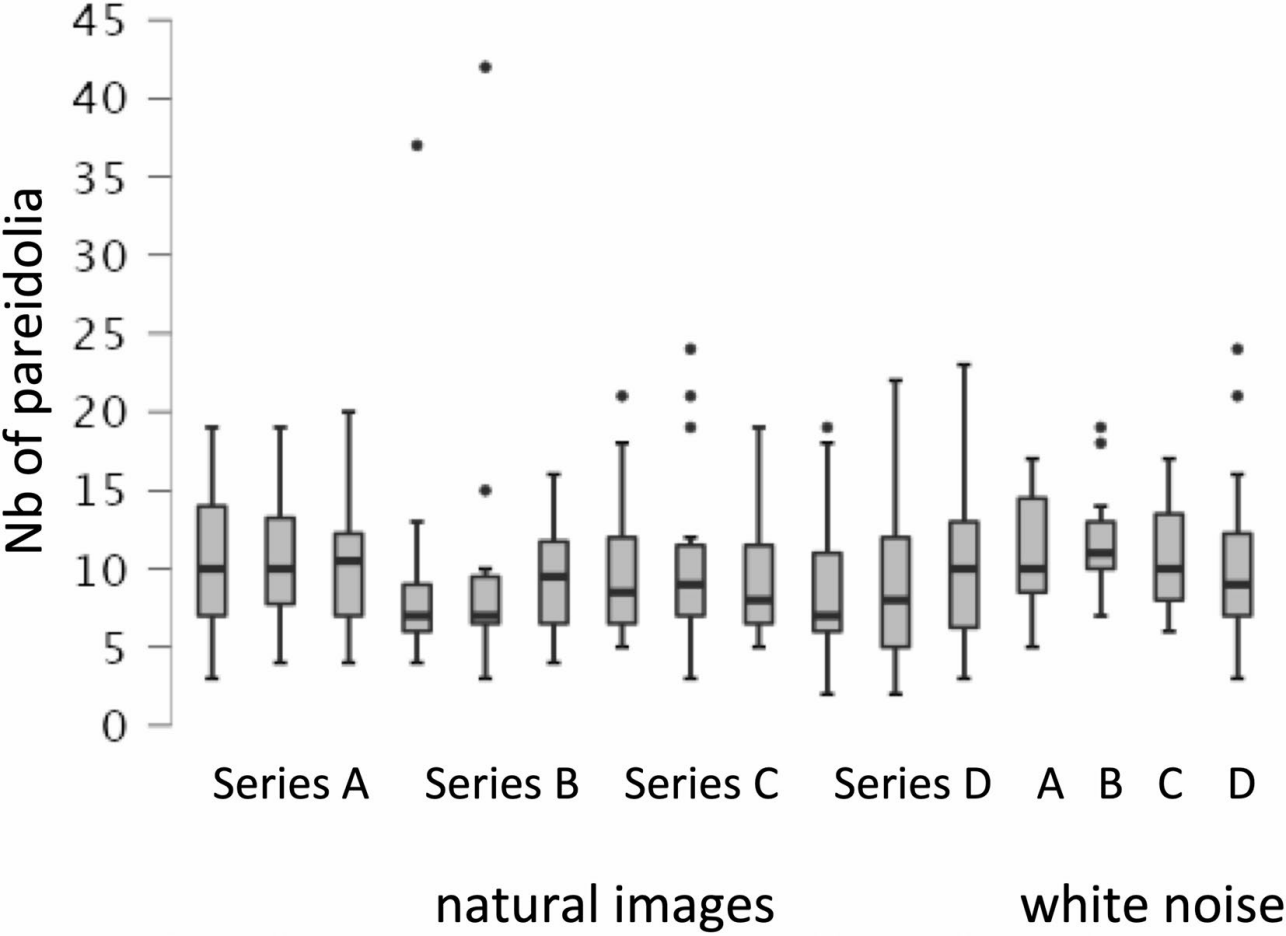
Boxplots showing the number of pareidolia produced across all 16 images (12 natural images (NI) and 4 white noise (WN) images). Kruskal–Wallis test revealed no significant difference between the number of pareidolia produced in response to NI and the WN images. Dots indicate outliers as defined by the boxplot convention based on the interquartile range (IQR). All data points were retained for analysis because pareidolia production varies substantially across individuals. Inspection of outlier cases showed that no participant was an outlier across all four images they viewed; two participants were outliers in two images, and all remaining outliers occurred in only one image per participant.

**Fig. 3:**
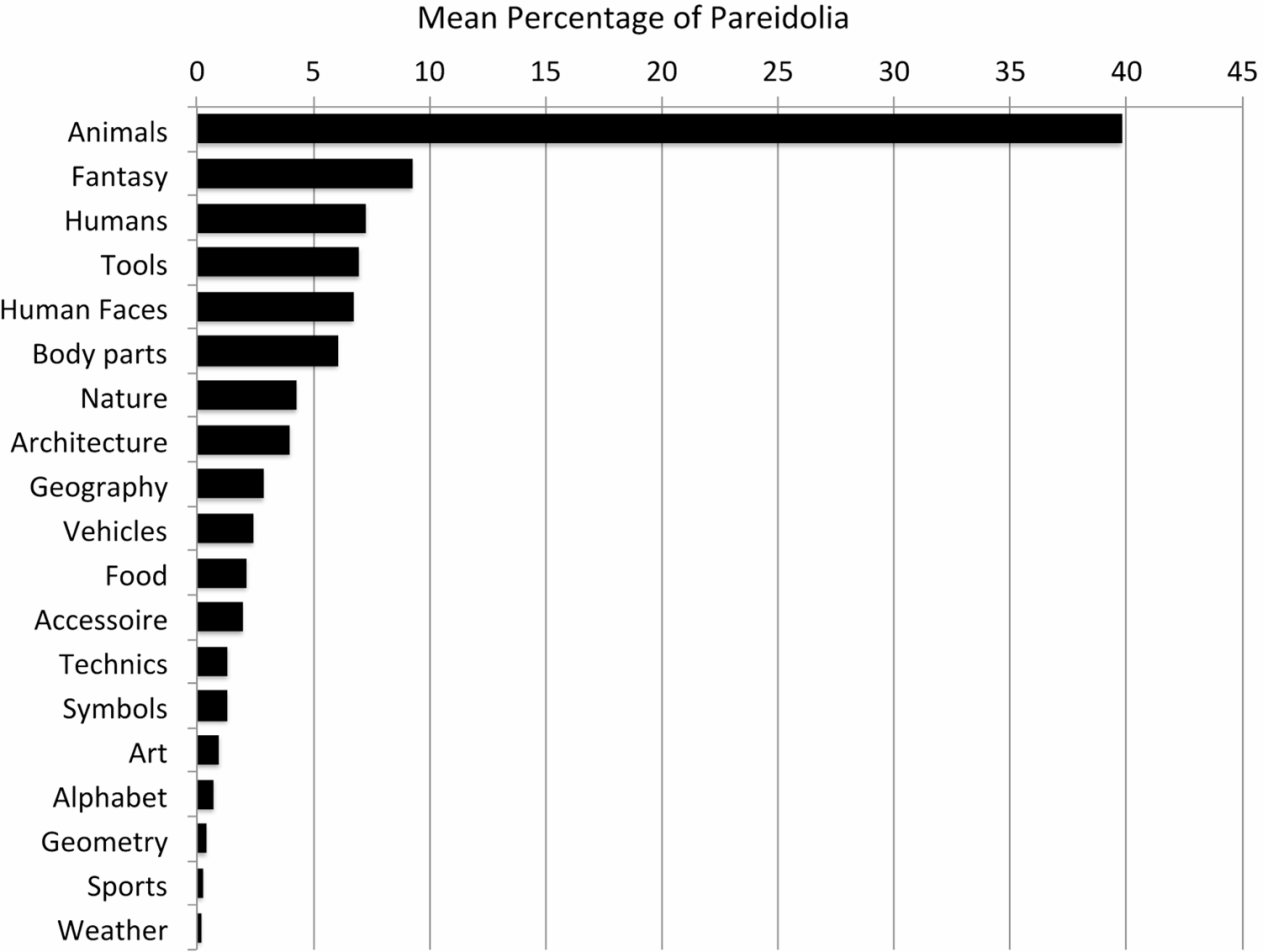
Mean percentages of pareidolia across the 19 defined semantic categories, sorted by frequency. The category “Animals” was the most common, accounting for nearly 40% of all pareidolia produced by the 81 healthy participants.

For each participant, the total number of pareidolia produced during the 5-min image presentation was counted (Fig. 2). The verbal responses provided by the participants were used for this analysis. These responses, originally given in Swiss-German, were translated into Standard German by two authors (N.G. and M.C.). Each pareidolia was then assigned to one of 19 semantic categories, following the classification scheme described in^14^ (for the complete list of categories, see Fig. 3). The category “Fantasy” encompassed non-realistic pareidolia—i.e., perceptions of imaginary, mythological, or fictional entities (e.g., monsters, dragons, fairies, dwarves, aliens, supernatural beings, fictional characters) that have no real-world referents. The number of pareidolia per image was compared between natural images (NI) and white noise images (WN) using a Kruskal–Wallis test.

To reduce dimensionality, we grouped these 19 categories into three superordinate clusters using GPT-4 (OpenAI)^22^, a large language model trained on diverse text data, including semantic relationships between objects and concepts^23^. The following prompt was provided to GPT-4: “Divide the following 19 terms (Technics, Geography, Tools, Geometry, Vehicles, Alphabet, Symbols, Nature, Food, Animals, Bodyparts, Human Faces, Humans, Accessories, Weather, Fantasy, Art, Sports, Architecture) into three clusters.” GPT-4 returned the following clusters, which were used for the subsequent analyses: Cluster 1: “Natural World”: Geography, Nature, Animals, Weather, Bodyparts, Human Faces, Humans; Cluster 2 “Human-Created Categories”: Technics, Tools, Vehicles, Accessories, Food, Sports, Architecture, Art; and Cluster 3 “Abstract Concepts”: Alphabet, Symbols, Geometry, Fantasy. The GPT-4 output is provided in Supplementary Material (S1). The percentage of pareidolia falling into each superordinate cluster was calculated for each participant.

All statistical analyses were conducted using JASP Version 0.16.1 (JASP Team, University of Amsterdam, 2022).

## Results

Median number of pareidolia production was 9 pareidolia per image during the image presentation time of 5 min (Interquartile range IQR: 5 pareidolia) with an individual range of 2 to 42 pareidolia (see Fig. 2). Kruskal–Wallis test revealed no significant differences in numbers of pareidolia produced in response to all the 16 images (H(15) = 20.642, *p* = 0.149), indicating that the different images as well as the different image types induced a similar number of pareidolia during the 5-min presentation period.

The frequency of pareidolia across the different semantic categories identified in this study is presented in Fig. 3. As shown, the distribution is skewed, with the semantic category “Animals” being the most common, accounting for nearly 40% of all pareidolia produced. This was followed by the categories “Fantasy”, “Humans”, “Tools”, “Human Faces”, and “Body parts”. The frequency of the remaining 12 categories was below than 5%.

In an additional analysis, we examined whether the six most common pareidolia categories (i.e., those appearing in more than 5% of responses: “Animal”, “Fantasy”, “Human”, “Tools”, “Human Faces”, and “Body arts”) showed systematic temporal patterns across the five-minute task. For each category, we calculated the percentage of occurrences at positions 1 through 14 and computed Pearson’s r between category frequency and pareidolia position. A significant correlation would indicate that a given category tends to appear earlier or later in the sequence.

**Fig. 4:**
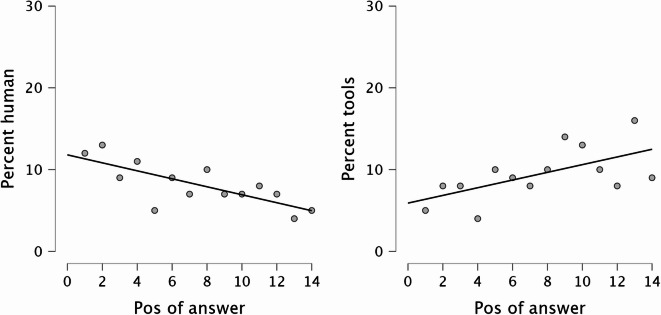
Temporal distribution of pareidolia categories. The occurrence of “Human” and “Tools” pareidolia showed significant correlations with pareidolia position within the five-minute task. “Human” pareidolia were more frequent earlier in the sequence (r = –0.760, *p* = 0.002), whereas “Tools” pareidolia increased in frequency later in the sequence (r = 0.610, *p* = 0.021). No other category showed a significant temporal trend.

This analysis revealed temporal dependencies for two categories: “Human” pareidolia occurred more frequently early in the sequence (r = -0.760, *p* = 0.002) whereas “Tool” pareidolia became increasingly common later on (r = 0.610, *p* = 0.021, see Fig. 4). In contrast, no significant temporal patterns were observed for “Animal”, “Fantasy”, “Human Faces”, and “Body parts” pareidolia (r = -0.022, *p* = 0.941; r = 0.013, *p* = 0.965; r = -0.086, *p* = 0.769; and r = 0.076, *p* = 0.797, respectively).

These findings indicate that pareidolia content is not randomly distributed over time. Instead, certain categories emerge preferentially at different stages, consistent with the idea that early pareidolia may rely more on rapid, perceptual mechanisms (e.g., detection of human figures), while later-emerging categories may reflect greater involvement of conceptual or imaginative processes.

**Fig. 5:**
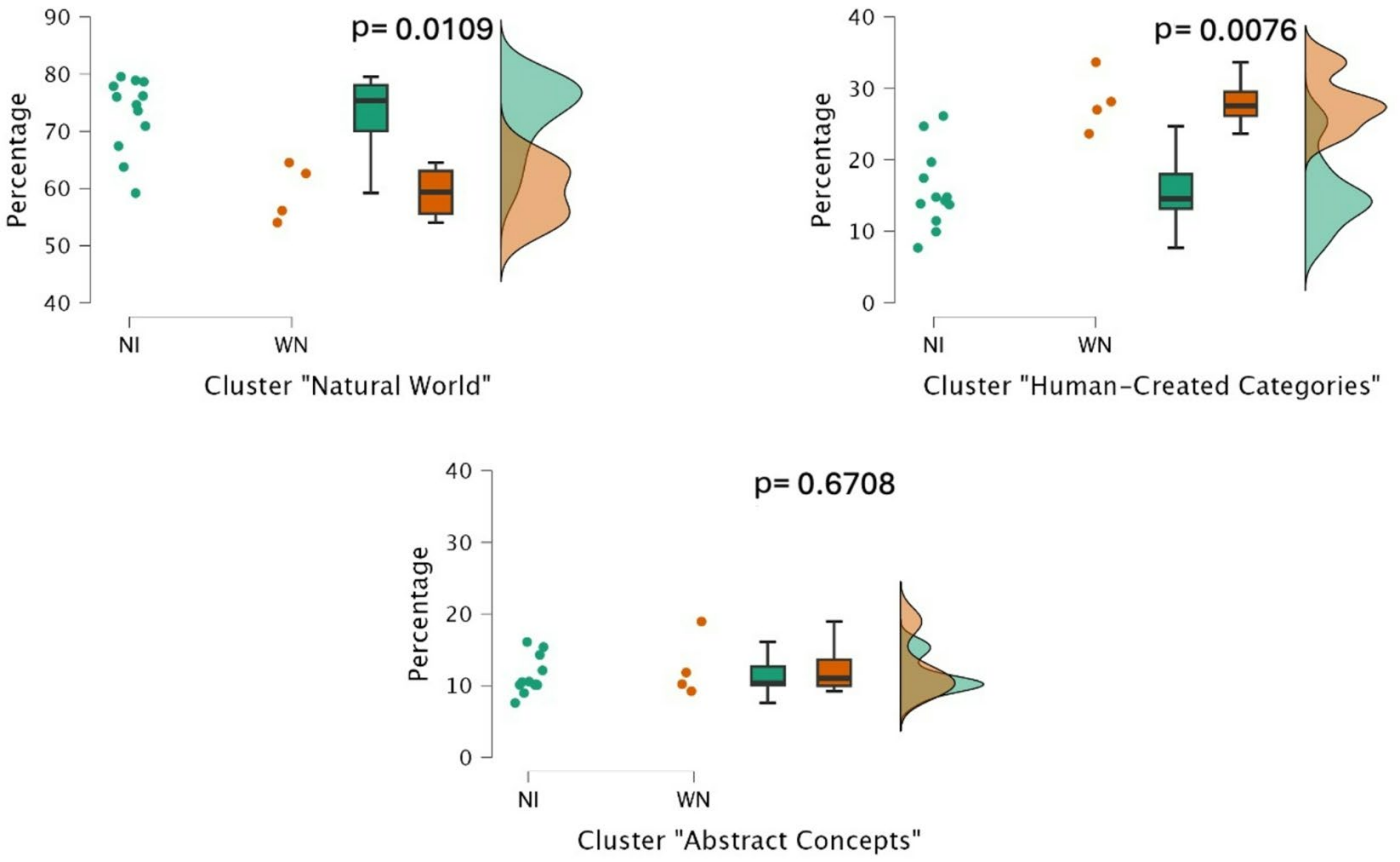
Raincloud plots illustrating the results for the three superordinate clusters. We found a dissociation in the frequency of pareidolia between NI and NW images dependent on the category. NI images prompted significantly more “Natural World” pareidolia compared to WN images (top line, left). Conversely, WN images evoked significantly more pareidolia in “Human-Created Categories” than NI images (top line, right). For “Abstract Concepts” cluster there was no significant difference between the two image types (bottom).

The statistical analysis of pareidolia percentages across the three superordinate clusters, as a function of image type, is shown in Fig. 5. For Cluster1, “Natural World”, Mann–Whitney test revealed a significant increase of the percentage of pareidolia found in NI images (median: 75.3%; Interquartile range (IQR): 8.04%) versus WN (median: 59.37%; IQR: 7.49%, U = 6.49; *p* = 0.0109). A reverse constellation was identified for Cluster2, “Human-Created Categories” with a significant decrease of these pareidolia in NI images (U = 7.118; *p* = 0.0076, median for NI: 14.53%, IQR: 4.84%; for WN median: 27.59%, IQR: 3.36%). Finally, for Cluster3, „Abstract Concepts “, there was no significant difference in percentage of pareidolia produced for NI (median: 10.35%, IQR: 2.57%) and WN (median: 11.02%, IQR: 3.65%; U = 0.181; *p* = 0.6708).

## Discussion

The objective of this study was to determine whether the number of pareidolia and the semantic categories of pareidolia are influenced by the type of images presented. To this end, we employed a dual-stimulus design with natural images (NI) and white noise images (WN). We found that the number of pareidolia did not differ between the two image types, but the image type significantly influenced the semantic categories of the pareidolia.

Our study differs from previous research on pareidolia in several key ways. Unlike most previous studies, our approach was not limited to identifying faces in the images. Instead, we allowed participants to engage with the images in a more open-ended manner, with a free instruction: “Look at the following images and let your imagination run free, draw everything you can see, except the objects themselves”. Additionally, we extended the duration of image presentation, allowing for a more comprehensive examination of the visual stimuli. In contrast to most previous studies, which typically presented images for a much shorter duration, we presented each image for five minutes. Our findings indicated that participants produced a median of nine pareidolia, with no significant differences between the two image types (i.e., WN and NI). This aligns with previous studies from our group, in which similar numbers of pareidolia were observed in samples of 50^1^ and 82 healthy participants^14^ using NI images and the same five-minute presentation time. While the overall number of pareidolia was consistent across conditions and studies, we found that participants showed considerable inter-individual variability in the number of pareidolia they produced.

The distribution of semantic categories was skewed under the open instruction condition, showing a preference for certain categories consistent with patterns described in previous studies^1,14^. The most frequently reported pareidolia were animals, which accounted for approximately 40% of all instances.

One explanation of this result is that the human ability to recognize animals is deeply rooted in evolutionary pressures that favoured survival and social interaction. Converging evidence from functional magnetic imaging (fMRI,^24^) and electroencephalographic (EEG,^25^) studies reveals a robust animal appearance bias in human visual processing, whereby the brain represents animal-shaped objects (zoomorphic items) more similarly to real animals than to inanimate objects, even when individuals consciously recognize them as non-living. As for face processing, this bias emerges early in visual processing, i.e., ~ 80–100 ms, through rapid feedforward mechanisms, likely reflecting an evolutionary adaptation for swift detection of potential threats or social agents. Moreover, a distinct temporal profile was proposed: an initial animal/face-like signal is typically followed at later latencies by neural activity reflecting accurate object categorization. This pattern suggests a two-stage process consisting of rapid appearance-based detection followed by feedback-driven identity verification^25^.

Bracci et al.^24^ demonstrated that within the ventral occipitotemporal cortex, representational patterns are strongly biased toward visual appearance. Objects with animal-like visual features cluster closely with real animals in neural representation space and are separated from other members of their actual object category. This organizational principle, which privileges animals and their visual features, may arise from both evolutionary constraints and individual learning history.

Behavioural evidence further supports this animal-specific processing bias. New et al.^26^ demonstrated that human visual attention incorporates a high-level, category-specialized system for continuous monitoring of animals. Using a change-detection paradigm, they found that participants were substantially faster and more accurate at detecting changes to animals compared to all other tested categories of inanimate objects. Critically, this animate monitoring advantage could not be explained by differences in low-level visual properties, familiarity, or expertise. These findings implicate evolved attentional mechanisms that prioritize objects based on their membership in ancestrally significant categories, independent of their contemporary relevance or utility. Such an attentional system allows for the rapid detection of animals in various environments, enhancing survival prospects. Importantly, non-human primates show similar object-recognition abilities, indicating that this trait is evolutionarily conserved and predates human-specific cultural developments^27^. Moreover, palaeolithic cave paintings predominantly depict animals^28^ and it has been suggested that pareidolia may be a key mechanism in Palaeolithic art making (e.g.,^29,30^).

Recent work shows that pareidolia is modulated by contextual factors, including the structure of the surrounding scene, the presence of competing face-like configurations, and the global arrangement of visual features. For example, pareidolic faces are detected more slowly when embedded among real faces, indicating competitive interactions between stimulus-driven templates^12^, and their attentional priority is shaped by contextual cues such as inversion or scene layout^9^. Our findings converge with this literature: natural images provide coherent visual structure that supports bottom-up grouping processes, leading to ‘Natural World’ interpretations, whereas the absence of visual structure in white noise promotes top-down, concept-driven pareidolia. Thus, the present results reinforce the view that pareidolia is not fixed, but dynamically shaped by stimulus context.

The brain therefore appears to employ two high-level specialized systems that prioritize the processing of human faces and animals, respectively. Both systems operate according to a sensitivity-over-specificity principle, whereby the system is optimized to minimize missed detections (false negatives) at the expense of occasional false alarms (false positives, i.e. pareidolia). Given that these systems likely operate competitively, their relative priority may shift depending on e.g., contextual factors, presentation duration, or task demands, leading either face or animal pareidolia to dominate perception under different conditions. However, the present data do not permit definitive conclusions regarding the relative contributions of these variables (see Limitations and Directions for Future Research below).

To investigate the impact of the image type (i.e., NI or WN) on pareidolia production, we employed GPT-4 to group the original 19 semantic categories into three superordinate clusters, i.e.: “Natural World”, "Human-Created Categories", and “Abstract Concepts”. The results indicated that the image type influenced the distribution of pareidolia across these clusters. Specifically, compared to WN images, NI elicited a significantly greater proportion of pareidolia belonging to the “Natural World” cluster. In contrast, pareidolia associated with the Human-Created Categories cluster were more frequently produced in response to WN images. No significant differences between NI and WN images were observed for pareidolia classified within the Abstract Concepts cluster.

Based on our data, we propose that the type of image serves as a modulator of the relative contribution of bottom-up and top-down processes. Specifically, for NI images, bottom-up mechanisms are more readily activated, leading to an increased production of “Natural World” pareidolia. In contrast, for WN images the contribution of bottom-up mechanisms is reduced. Instead, top-down processes driven by semantic knowledge, particularly concepts stored in the mental lexicon and general knowledge, may play a more prominent role, thereby facilitating the emergence of “Human-Created Categories” pareidolia.

### Limitations and directions for future research

Using highly unconstrained tasks with long presentation times creates more naturalistic testing conditions, allowing participants to engage freely with the stimuli. However, this realism comes with a cost: such designs make it difficult to control the many factors that may influence participants’ behavior, thereby potentially compromising internal validity. A key challenge is that participants may generate their own “intrinsic” instructions when tasks are loosely constrained and extend over longer durations. Thus, while unconstrained designs enhance ecological validity, they simultaneously introduce confounds that make causal inference more difficult to interpret.

Natural images and white noise stimuli differ in both spatial structure (including spatial-frequency distribution) and chromatic composition, making it difficult to isolate which specific image features drive the observed between-condition differences. Future studies could address this by employing intermediate stimulus classes—such as fractal noise or phase-scrambled derivatives of the natural images—which would allow a more systematic dissociation of low-level visual properties (often referred to as bottom-up cues).

While our findings reveal robust differences between image types, future studies using such controlled stimulus manipulations – alongside variations in presentation time and task constraints—will be needed to disentangle these influences and develop a more complete mechanistic account of the competitive dynamics between face- and animal-selective processing systems.

## Conclusions

The objective of this study was to investigate the influence of NI and WN on the perception of pareidolia. Both types of images resulted in an equivalent frequency of pareidolia during the five-minute presentation time, suggesting that the human brain is predisposed to perceive meaningful patterns, irrespective of the perceptual stimulus. We found that the most frequent form of pareidolia is the perception of animals, which may be explained by evolutionary pressures. The dissociation between the number of pareidolia in the NI for the cluster “Natural World” and the number of pareidolia in WN images in the cluster "Human-Created Categories" indicates different mechanisms involved such as Gestalt psychology principles and semantic knowledge. Finally, our study demonstrates that the image selection impacts the type of pareidolia produced, highlighting the importance of the experimental image when designing experiments with open instructions.

## Supplementary Information

Supplementary Information.

## Data Availability

The data supporting the conclusions of this study will be made available by the corresponding author upon reasonable request.
