# Supplementary material for: Image type reveals evolutionarily shaped perceptual and conceptual mechanisms of pareidolia
Source: Sci Rep. 2026 Apr 15;16:17606. doi: 10.1038/s41598-026-47242-x (PMC13243614; doi:10.1038/s41598-026-47242-x)
Supplement: Supplementary file 1 — Supplementary Information. [file 41598_2026_47242_MOESM1_ESM.pdf]

Supplementary Material

S1. Semantic Clustering Procedure

The following prompt was used to generate the semantic clustering applied in the analysis:

“Divide the following 19 terms (Technics, Geography, Tools, Geometry, Vehicles, Alphabet, Symbols, Nature, Food, Animals, Bodyparts, Human Faces, Humans, Accessories, Weather, Fantasy, Art, Sports, Architecture) into three clusters.”

GPT-4 Output (Clustering Solution)

GPT-4 provided the following clustering, which was used in the subsequent analyses:

- Cluster 1: Natural World
    - Geography
    - Nature
    - Animals
    - Weather
    - Bodyparts
    - Human Faces
    - Humans
- Cluster 2: Human-Created Categories
    - Technics
    - Tools
    - Vehicles
    - Accessories
    - Food
    - Sports
    - Architecture
    - Art
- Cluster 3: Abstract Concepts
    - Alphabet
    - Symbols
    - Geometry
    - Fantasy
